# Connecting Mechanics and Bone Cell Activities in the Bone Remodeling Process: An Integrated Finite Element Modeling

**DOI:** 10.3389/fbioe.2014.00006

**Published:** 2014-04-08

**Authors:** Ridha Hambli

**Affiliations:** ^1^Prisme Institute, Polytechnique Orleans, PRISME/MMH, Orleans, France; ^2^I3MTO, Université d’Orléans, Orleans, France

**Keywords:** finite element, bone remodeling, mechanical stimulus, basic multicellular unit, proximal femur

## Abstract

Bone adaptation occurs as a response to external loadings and involves bone resorption by osteoclasts followed by the formation of new bone by osteoblasts. It is directly triggered by the transduction phase by osteocytes embedded within the bone matrix. The bone remodeling process is governed by the interactions between osteoblasts and osteoclasts through the expression of several autocrine and paracrine factors that control bone cell populations and their relative rate of differentiation and proliferation. A review of the literature shows that despite the progress in bone remodeling simulation using the finite element (FE) method, there is still a lack of predictive models that explicitly consider the interaction between osteoblasts and osteoclasts combined with the mechanical response of bone. The current study attempts to develop an FE model to describe the bone remodeling process, taking into consideration the activities of osteoclasts and osteoblasts. The mechanical behavior of bone is described by taking into account the bone material fatigue damage accumulation and mineralization. A coupled strain–damage stimulus function is proposed, which controls the level of autocrine and paracrine factors. The cellular behavior is based on Komarova et al.’s (2003) dynamic law, which describes the autocrine and paracrine interactions between osteoblasts and osteoclasts and computes cell population dynamics and changes in bone mass at a discrete site of bone remodeling. Therefore, when an external mechanical stress is applied, bone formation and resorption is governed by cells dynamic rather than adaptive elasticity approaches. The proposed FE model has been implemented in the FE code Abaqus (UMAT routine). An example of human proximal femur is investigated using the model developed. The model was able to predict final human proximal femur adaptation similar to the patterns observed in a human proximal femur. The results obtained reveal complex spatio-temporal bone adaptation. The proposed FEM model gives insight into how bone cells adapt their architecture to the mechanical and biological environment.

## Introduction

Bone remodeling is a dynamic process in which old bone is removed by osteoclasts and new bone is added by osteoblasts. Osteoblasts produce inorganic calcium phosphate, which is converted to hydroxyapatite, and an organic matrix consisting mainly of type I collagen, and then they deposit new bone on the part of the bone resorbed by osteoclasts. Osteoclasts dissociate calcium by secreting acid and degrade organic components by releasing lysosomal enzymes. Interactions between osteoblasts and osteoclasts are critical in the regulation of bone remodeling. These coupled activities take place in a basic multicellular unit (BMU) (Frost, 2001) and are modulated by mechanical and biological factors (Komarova et al., 2003). It is well-known that the development and activity of osteoclasts are under the control of the osteoblasts (Rodan and Martin, 1981; Hambli and Rieger, 2012).

Disruptions in bone remodeling contribute to the pathogenesis of disorders such as osteoporosis and Paget’s disease. Therefore, in order to develop more appropriate mechanobiological computer models to simulate the bone remodeling process, it is necessary to incorporate the combined effects of bone cell activities and the mechanical behavior of bone.

Several categories of bone remodeling models have been proposed by different authors: models based on the global optimality criterion (Hollister et al., 1994; Bagge, 2000; Tovar et al., 2006; Jang and Kim, 2008, 2010), models based on maintaining a homeostatic state of stress/strain/strain energy (Carter et al., 1987, 1989; Huiskes et al., 1987; Beaupré et al., 1990; Prendergast and Taylor, 1994; Martin, 1995; Hart and Fritton, 1997; Jacobs et al., 1997; Fernandes et al., 1999; Ruimerman et al., 2005; Hambli et al., 2009, 2011; Adachi et al., 2010; Hambli, 2011), models based on damage accumulation/repair (Prendergast and Taylor, 1994; Martin, 1995; Ramtani and Zidi, 2001; McNamara and Prendergast, 2007), mechanistic models considering both mechanical and metabolic factors in the remodeling loop (Hernandez et al., 2000, 2001; Huiskes et al., 2000; Hazelwood et al., 2001; Taylor and Lee, 2003; Taylor et al., 2004; Aznar et al., 2005), and a recent model considering the interstitial fluid flow (Tsubota et al., 2009). Some authors have developed simple 2D finite element (FE) remodeling simulation based on a reaction–diffusion system influenced by mechanical stress (Matsuura et al., 2002, 2003; Tezuka et al., 2003, 2005). These continuum models have achieved some success in predicting normal bone architecture. They have however a major deficiency. They use mechanical stress or strain as a control system in which bone functional adaptation is driven by the error between a mechanical set point and a mechanical stimulus to predict bone remodeling behavior, without considering the biophysical activities of osteoblasts and osteoclasts.

There are currently a limited number of mathematical models, which describe the activity of BMUs. The work by Komarova et al. (2003) was the first to model mathematically the non-linear autoregulation between osteoblasts and osteoclasts by expressing autocrine and paracrine factors. Rattanakul et al. (2003) proposed a model considering PTH as the main regulatory element in bone formation and resorption. In his work, Moroz et al. (2006) proposed a dynamic model that includes Michaelis–Menten type of feedback mechanisms. Lemaire et al. (2004) developed a more sophisticated model to describe the explicit molecular interaction and autoregulation between osteoblasts and osteoclasts. This model includes the well-known cytokine receptor activator of nuclear factor κB (RANK), its ligand (RANKL), and osteoprotegerin pathway (OPG) (RANK/RANKL/OPG), PTH, and also transforming growth factor β (TGFβ). Based on Lemaire et al. (2004), Maldonado et al. (2006) built a model, which takes the influence of the osteocyte under mechanical stimulation into account. Pivonka et al. (2008) subsequently developed a model, which also exhibits the RANK/RANKL/OPG pathway PTH and TGFβ but is based on Hill functions, which are better suited to express the binding mechanism between ligand and receptor. Finally, the recent model by Ryser et al. (2009) provides enhanced modeling of autocrine and paracrine factors following Komarova’s model (2003). Based on the spatio-temporal dynamic observation of BMU behavior, it also includes the explicit description of the RANK/RANKL/OPG pathway. A review of the previous BMUs models shows that (i) the Komarova, Moroz, and Rattanakul models are simple but need a limited number of parameters (8–2) and (ii) the Lemaitre, Pivonka, and Ryser models are more sophisticated but need a significantly higher number of parameters (>90). While these cell-based models give theoretical insight into bone regulation mechanisms including metabolic factors, none of these models were implemented into an FE codes to simulate the bone remodeling process from a mechanobiological perspective considering the cells activities’ interactions with the mechanical reaction of bone.

In current work, an extension of Komarova et al. (2003) model was implemented into an FE code to simulate the remodeling process from a mechanobiological point of view.

The bone adaptation approach used in this study allows for the computation of changes in bone mass at a discrete site of bone remodeling at a macroscopic scale. The modeling of these interactions with biological factors suggested by Komarova et al. (2003) was completed by Bonfoh et al., 2011) who considered the influence of an external loading (stimulus effects on the bone cell dynamics). We combined Komarava et al.’s model and that of Bonfoh et al. (2011) (i) to include more general mechanical behavior of the bone such as fatigue damage growth and repair, mineralization, porosity, and bone material properties evolution and (ii) to include the principle of cellular accommodation, suggesting that the reference stimulus value for bone remodeling activation is not constant, but dependent on the load history. In addition, a sensitivity analysis (SA) was performed to investigate the impact of the model factors’ sensitivities on the predicted bone density of a selected region of interest (ROI) (femur neck).

The focus here was to develop and test the mechanobiological remodeling algorithm rather than to investigate the remodeling process of a real 3D proximal femur and/or develop a parametric study of the role of the remodeling factors on bone density variations. The predictive potential of the current model enables one to investigate the effect of bone cell rate changes combined with mechanical external loads. Specifically, the model may offer a computer simulation framework to explore the development of new therapeutic treatments to pathological conditions and bone disorders such as osteoporosis.

## Remodeling Model Description

During bone remodeling, the applied external load is transmitted in the form of stress/strain to the local bone site. Then the mechanical signals (stimuli) are received by osteocytes, which subsequently stimulate osteoclast and osteoblast populations in BMUs to change the bone mass.

**Figure 1:**
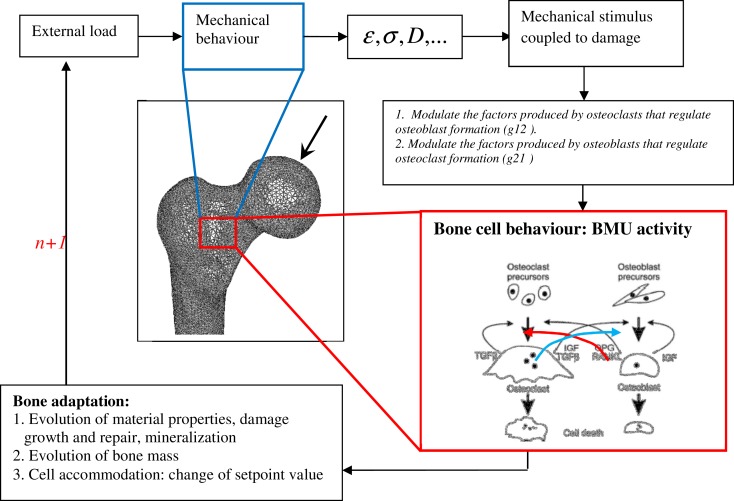
**Schematic representation of bone remodeling based on BMU activity coupled to mechanical stimulus: at the remodeling cycle (*n*), the applied load generates mechanical stress, strain, and fatigue damage states at every FE of the mesh**. A stimulus is then sensed by osteocytes at every bone site. The stimulus is converted into signals, which control the osteoblast and osteoclast interactions. Bone formation and removal is performed by competition between osteoblast and osteoclast growth at the given bone site.

The corresponding mechanobiological remodeling algorithm is illustrated in Figure 1. The model was implemented in the Abaqus code (UMAT subroutine) using a time step of 1 day.

### Mechanical behavior

To describe the continuum mechanical behavior of bone during the remodeling process considering the fatigue damage effects, the concept of continuum damage mechanics (CDM) can be used. In this case, the behavior law coupled to damage can be expressed by Hambli (2010), Hambli and Thurner, 2013):

(1) $$\sigma_{\text{ij}}=\left(1-D^{\text{fat}}\right)a_{\text{ijkl}}\varepsilon_{\text{kl}}$$

where σ_ij_ is stress, *D*^fat^ is the fatigue damage variable, ε_kl_ is strain, and *a*_ijkl_ is the isotropic elasticity stiffness tensor.

For high cycle fatigue under purely elastic strain, Chaboche (1981) proposed a non-linear damage model given by:

(2) $$D^{\text{fat}}=1-{\left[1-{\left(\frac{N}{N_{f}}\right)}^{\frac{1}{1-\gamma }}\right]}^{\frac{1}{1+\beta }}$$

where γ and β are material parameters and *N*_f_ is the cycle at failure, which can be obtained as described by Martin et al. (1998):

(3a) $$N_{f}^{c}=1.479\times 10^{-21}\Delta \varepsilon^{-10.3}\text{ for compressive loads}$$

(3b) $$N_{f}^{t}=3.630\times 10^{-32}\Delta \varepsilon^{-14.1}\text{ for tensile loads}$$

where Δε is the amplitude of the applied microstrain.

Combining damage definition and elastic modulus evolution, the elastic modulus *E* at each location is calculated from the density ρ[computed using Komarova’s model based on Eq. (12 and 17)], the bone mineralization and the damage according to Hambli et al. (2009):

(4) $$E=C\left(1-D^{\text{fat}}\right)\rho^{p}\alpha^{q}$$

where *C* is experimentally derived constants (2 ≤ *p* ≤ 3) and *q* = 2.74 (Hernandez et al., 2001).

α is the ash function, which denotes the degree of bone mineralization expressed by Hernandez et al. (2000):

(5) $$\alpha \left(t\right)=\alpha_{\max}+\left(\alpha_{0}-\alpha_{\max}\right)e^{-\lambda t}$$

α_0_, α_max_, and *k* denote initial mineralization, maximum degree of mineralization, and a parameter determining the shape of the temporal evolution curve. The average value for α_0_ is about 0.65 (Martin et al., 1998) with 0 ≤ α ≤ α_max_ = 1.

Apparent bone porosity *p* can be directly approximated by Hernandez et al. (2000, 2001):

(6) $$p=1-\frac{\rho }{1.41+1.29\alpha }$$

### Mechanical stimulus

Various expressions of the mechanical stimulus involved in bone remodeling have been proposed in the literature. The mechanical stimulus used here is expressed in terms of strain energy density. Therefore, the mechanical signal sensed by an osteocyte *k* at its location *x*_k_ is given by Mullender and Huiskes (1995):

(7) $$S\left(x,\;t\right)\;=\sum_{k=1}^{N_{\text{oc}}}f_{k}\left(x\right)\mu_{k}\left(S_{k}-\bar{S_{k}}\right)$$

where μ_k_ is the mechanosensitivity of the osteocyte *k* and *N*_oc_ is the number of osteocytes. ${\bar{S}}_{k}$ is the threshold value of the signal considering that an equilibrium state can be obtained for near the reference value, ${\bar{S}}_{k}$. Far from this value, unbalanced activity of osteoblasts and osteoclasts is observed, which leads to bone apposition or resorption.

*f_i_*(*x*) is a spatial influence function defined by Mullender and Huiskes (1995):

(8) $$f_{k}\left(x\right)\;=\text{exp}\left(-d_{k}\left(x\right)∕d_{0}\right)$$

where *d_k_*(*x*) is the distance between the osteocyte *k* and the bone surface location *x*. The parameter *d*_0_ is a normalization factor that limits the area of influence of the osteocyte.

*S_k_* is the local stimulus value expressed in terms of coupled strain–damage energy density and is expressed by:

(9) $$S_{k}=\frac{1}{2}\left(1-D^{\text{fat}}\right)\sigma_{\text{ij}}\varepsilon_{\text{ij}}$$

Osteocytes respond to low bone deformation (disuse) or fatigue-related microfractures by activating osteoclasts to resorb locally the bone matrix. This phase is followed by a period of bone formation by osteoblasts. Hence, mechanotransduction is a stimulatory signal to osteoblasts and an inhibitory signal to osteoclasts.

This stimulus function coupled to damage ensures damage repair. When the fatigue damage reaches its critical value at fracture (*D*^fat^ ≈ 1) at a given bone site, the stimulus is set to zero. Therefore, the osteoclasts are activated to resorb the damaged bone zone.

In addition, it is well-known that physical activity causes relatively larger changes in bone mass and strength in young people than in adults (Kassem et al., 1996), suggesting that the loss of bone in the aging may be attributed to a reduced sensitivity of bone setpoints to the mechanical stimulus sensed by bone cells (Turner, 1999; Frost, 2001) introduced the principle of cellular accommodation based on bone stimulus setpoint changes. He hypothesized that the stimulus setpoints are not constant, but are dependent on the load history, which can modify their values in an adaptive way resulting from the transient nature of many cellular biochemical responses to mechanical loading. Variation of the setpoints with time (aging) can be expressed by Schriefer et al. (2005):

(10) $${\bar{S}}_{k}=S_{k}^{0}+\left(S_{k}-S_{k}^{0}\right)\left(1-e^{-\lambda t}\right)$$

$S_{k}^{0}$ denotes the initial setpoint value and the parameter λ controls the velocity of the adaptation.

### Bone cells dynamic behavior

Bone remodeling involves bone resorption by osteoclasts followed by the formation of new bone by osteoblasts. During bone remodeling, osteoclasts and osteoblasts interact with each other by expressing autocrine and paracrine factors that regulate the cell population. In the current work, a bone remodeling model developed by Komarova et al. (2003) to describe the dynamics of cell populations at a remodeling site has been implemented into the FE code Abaqus. In this model, the osteoblast and osteoclast cell growth rates are described in the form of two differential equations regulated by autocrine and paracrine interactions. Autocrine signaling represents the feedback from osteoclasts and osteoblasts to regulate their respective formation. Paracrine signaling represents the factors produced by osteoclasts that regulate osteoblast formation, and vice versa. Among the multitude of biochemical factors, only OPG/RANK/RANKL and TGFβ/IGF pathways were modeled implicitly by Komarova et al. (2003) in the form of non-linear interactions between osteoclasts and osteoblasts populations.

The system of differential equations describing the osteoclast and osteoblast rates and interactions using parameters, which characterize the autocrine and paracrine factors can be expressed by:

(11) $$\left\{\begin{array}{c} \frac{dx_{B}}{dt}=\alpha_{2}x_{C}^{g12}x_{B}^{g22}-\beta_{2}x_{B}\;\\ \frac{dx_{C}}{dt}=\alpha_{1}x_{C}^{g11}x_{B}^{g21}-\beta_{1}x_{C}\; \end{array}\right.$$

where *x*_C_ and *x*_B_ denote, respectively, the osteoclast and osteoblast populations.

α_1_ is the osteoclast production rate, β_1_ is osteoclast removal rate, α_2_ is the osteoblast production rate, β_2_ is the osteoclast removal rate.

Parameter *g*11 describes the combined effects of all the factors produced by osteoclasts that regulate osteoclast formation (osteoclast autocrine regulation).

Parameter *g*22 describes the combined effects of all the factors produced by osteoblasts to regulate osteoblast formation (osteoblast autocrine regulation).

Parameter *g*12 describes the combined effects of all the factors produced by osteoclasts that regulate osteoblast formation, such as TGFβ (osteoclast-derived paracrine regulation).

Parameter *g*21 describes the combined effects of all the factors produced by osteoblasts that regulate osteoclast formation, such as OPG and RANKL (osteoblast-derived paracrine regulation).

The model assumes that osteoclast and osteoblast apoptosis is not affected by additional autocrine/paracrine regulators. Therefore, the decay terms of osteoclasts and osteoblasts are linear.

The variation in bone density ρ at the remodeling site is expressed in terms of percentage of the initial mass depending on the number of osteoclasts and osteoblasts:

(12) $$\frac{d\rho }{dt}=k_{2}X_{B}-k_{1}X_{C}$$

where *K*_1_ and *K*_2_ are the normalized activities, *X*_c_ and *X*_B_ are, respectively, the numbers of actively resorbing osteoclasts and forming osteoblasts at a remodeling site defined by Komarova et al. (2003):

(13) $$\left\{ \begin{array}{l} X_{C}=x_{C}-{\bar{x}}_{C}\;if\;x_{C}>{\bar{x}}_{C}\\ X_{C}=0\;if\;x_{C}\leq {\bar{x}}_{C} \end{array}\right.$$

and

(14) $$\left\{ \begin{array}{l} X_{B}=x_{B}-{\bar{x}}_{B}\;if\;x_{B}>{\bar{x}}_{B}\\ X_{B}=0\;if\;x_{B}\leq {\bar{x}}_{B} \end{array}\right.$$

Where ${\bar{x}}_{C}$ and ${\bar{x}}_{B}$ are, respectively, the number of osteoclasts and osteoblasts at steady state expressed by Komarova et al. (2003):

(15) $$\left\{\begin{array}{c} {\bar{x}}_{B}={\left(\frac{\beta_{1}}{\alpha_{1}}\right)}^{\frac{g12}{\gamma }}{\left(\frac{\beta_{2}}{\alpha_{2}}\right)}^{\frac{\left(1-g11\right)}{\gamma }}\;\\ {\bar{x}}_{C}={\left(\frac{\beta_{1}}{\alpha_{1}}\right)}^{\frac{\left(1-g22\right)}{\gamma }}{\left(\frac{\beta_{2}}{\alpha_{2}}\right)}^{\frac{g21}{\gamma }}\; \end{array}\right.$$

where

(16) $$\gamma =g12g21-\left(1-g11\right)\left(1-g22\right)$$

The new density value of the bone tissue is approximated using the forward Euler method by:

(17) $$\rho_{t+\Delta t}=\rho_{t}+\Delta \rho$$

During remodeling cycles, mechanical signals received by osteocytes stimulate pre-osteoclast and pre-osteoblast populations, which convert the signals into autocrine and paracrine factors (gij). These factors stimulate and regulate osteoclast and osteoblast populations, which compete in BMUs to change bone mass.

In this paper, we consider the particular condition where a bone cell grows normally and only influences its neighbor’s activity, but does not produce autocrine factors (*g*11 = *g*22 = 0). Therefore, the signal received by osteoclasts and osteoblasts influences the autocrine and paracrine factors’ productions through the exponents, *g*_ij_ by Bonfoh et al. (2011):

(18) $$\left\{\begin{array}{c} g11=g22=0\;\\ g12=A_{1}+B_{1}e^{-\gamma_{1}S\left(x,\;t\right)}\;\\ g21=A_{2}+B_{2}e^{-\gamma_{2}S\left(x,\;t\right)}\;\\ \; \end{array}\right.$$

A_1_, B_1_, A_2_, B_2_, γ_1_, and γ_2_ are model parameters that regulate the production of paracrine factors *S*(*x, t*) denotes the mechanical stimulus function.

The BMUs in cortical and trabecular bone have the same biological structure but different morphological organizations. The remodeling or renewal of bone tissue constitutes 25% of trabecular and 2–3% of cortical bone renewal each year (up to 10 times higher for trabecular bone) (Parfitt, 1994; Rho et al., 1998). Therefore, to distinguish between BMU activity in cortical and trabecular bone, the Komarova model was applied for both bones but with a bone density (Eq. 12) 10 times lower for cortical bone ($k_{i}^{\text{corti}}=0.1\times k_{i}^{\text{trab}}i=1,\;2$).

The model parameters are summarized in Table 1.

**Table 1:** **Material properties for bone used for the remodeling simulation from Mullender and Huiskes (1995), Hernandez et al. (2001), Komarova et al. (2003), Hambli et al. (2009)**.

Parameters			Notation	Trabecular bone	Cortical bone
**GENERAL PARAMETERS**					
Initial elastic modulus			*E*_0_ (MPa)	2000	17000
Poisson ratio			ν	0.3	0.3
Initial density			ρ (g/cm^3^)	0.764	1.4
Density coefficient			*C* (g/cm^3^)	4000	80003
Density exponent			*P*	3	3.
Ash exponent			*Q*	2.74	2.74
**DAMAGE LAW PARAMETERS**					
Fatigue parameter			γ	0.2	0.2
Fatigue exponent			β	0.4	0.4
**MINERALIZATION PARAMETERS**					
Initial ash fraction			α_0_	0.6	0.6
Maximum physiological value			α_max_	0.7	0.7
Velocity of the mineralization			*k* (days^−1^)	0.0003387	0.0003387
**STIMULUS PARAMETERS**					
Mechanosensitivity of the osteocyte			μ*_k_* (nmol mm J^−1^ h^−1^)	0.5	0.5
Osteocytes density			*N*_oc_ (mm^−3^)	10625	10625
Spatial influence factor			*d*_0_ (μm)	0.1	0.1
Accommodation velocity parameter			λ (days^−1^)	0.002	0.002
Initial setpoint value			$S_{k}^{0}\left(\text{J}{\text{m}}^{-3}\right)$	0.0025	0.0025
**BMU PARAMETERS**					
**Osteoclasts**			**Osteoblasts**		
**Notation**	**Trabecular**	**Cortical**	**Notation**	**Trabecular**	**Cortical**

α_1_ (osteoclasts/day)	3	3	α_2_ (osteoblasts/day)	4	4
β_1_ (osteoclasts/day)	0.2	0.2	β_2_ (osteoblasts/day)	0.0017	0.0017
*k*_1_ (osteoclasts/day)	0.24	0.024	*k*_2_ (osteoblasts/day)	0.02	0.002
*A*_1_	1.6	1.6	*A*_1_	−1.6	−1.6
*B*_1_	−0.49	−0.49	*B*_2_	0.6	0.6
γ_1_ (g/J)	16.67	16.67	γ_2_ (g/J)	33.37	33.37
*x*_C_ (*t* = 0) (osteoclasts)	15	15	*x*_B_ (*t* = 0) (osteoblasts)	1	1

### Simulation of femoral head remodeling

**Figure 2:**
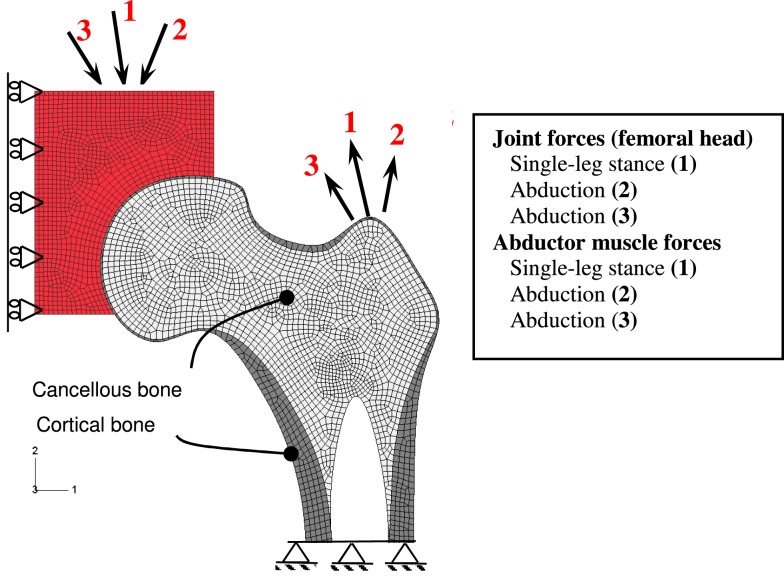
**FE of the proximal femur and boundary conditions**.

To illustrate the capabilities of the mechanobiological bone adaptation model developed, remodeling of a 2D proximal femur was performed. The 2D model is based on the geometry of a real femur, taken from a radiograph of a coronal section of the proximal femur (Jacobs et al., 1995). An FE model was constructed including the trabecular and cortical bones and a representation of the acetabulum allowing for the free femur head articulation (Figure 2). The purpose of the model is to show the adaptation of the proximal femur only (trabecular and cortical bone). Hence, the acetabulum appears as an exterior element, which participates in load bearing, but is not part of the adaptation process in the algorithm.

A 2D mesh was generated using four-node plane stress elements. Although clinical observations provided valuable information regarding the changes of cortical and trabecular bone types during growth or aging, little is known about how each of the cortical and trabecular bone types changes and how they interact with each other during the bone remodeling process (Jang and Kim, 2010). Thus, thresholding between cortical and cancelous bone is modeled by two bone regions with the same mechanical and BMU behavior laws but with different material properties.

The remodeling cycle chosen here is characterized by loads representing a daily gait cycle (Figure 2). The daily loading history was simulated by three load cases consisting of joint reaction and abductor muscle forces similar to those proposed by Carter et al. (1987) for normal activity. Because it takes 3–4 months for one remodeling cycle to complete the sequence of bone resorption, formation, and mineralization (Mundy, 1999), a minimum of 6–8 months is required to achieve a new steady-state bone mass that is measurable. In the present work, the simulation was run for 5 years (1825 days) under normal daily loading, leading to redistribution of the density and remodeling parameters (baseline) condition.

To illustrate the potential of the current integrated FE model, three remodeling analyses were performed corresponding to three cases of applied forces (Table 2):

(a) Case NFC refers to a normal force case (standard walking gait) (Carter et al., 1987).
(b) Case LFC refers to a low force case (10% of the force of case NFC).
(c) Case HFC refers to a high force case (150% of the force of case NFC).

**Table 2:** **Selected load conditions that simulate walking under three different conditions**.

Remodeling load case	Cycles/day	Femoral head (*N*)	Orientation (°) FP	Abductor muscle forces (*N*)	Orientation (°) FP
Low force case (LFC) (LFC = 0.1 × NFC)	6000	(1) 232	24	(1) 70	28
	2000	(2) 116	−15	(2) 35	−8
	2000	(3) 155	56	(3) 47	35
Normal force case (NFC)	6000	(1) 2317	24	(1) 703	28
	2000	(2) 1158	−15	(2) 351	−8
	2000	(3) 1548	56	(3) 468	35
High force case (HFC) (HFC = 1.5 × NFC)	6000	(1) 3244	24	(1) 984	28
	2000	(2) 1621	−15	(2) 491	−8
	2000	(3) 2167	56	(3) 655	35

*Case NFC refers to the normal force case (standard walking gait) (Carter et al., 1987). Case LFC refers to the low force case (10% of the force of case NFC) and case HFC refers to the high force case (150% of the force of case NFC). The model consists of cortical (420 elements) and trabecular (1350 elements) and acetabulum (570 elements) regions. The femur is fixed at the bottom and loaded through the acetabulum and abductor muscle forces (details in Table 2) (Carter et al., 1987). Variable cortical bone thickness (dark gray), cancelous bone (light gray), and acetabulum (red)*.

A very low value of applied force was used for case LFC (the standard walking gait loads were reduced by 90%) to generate a large disuse femur region to test the potential of the algorithm to perform bone remodeling in sites where the level of mechanical stress is low or non-existent. Such very reduced loads concern patients subjected to long bed rests or can be considered as the effects of microgravity on bone due to long-duration space flight for astronauts.

The Femur model was run in alternating load and unload (*F* = 0 N) increments for 1825 iterations (days) with a fixed number of cycles per day and orientations of forces (Table 2).

Other inputs can influence the remodeling response (frequency, age, drugs, etc.). The aim of the current work was to implement the novel remodeling FE model and to check its validity to predict bone adaptation processes under different conditions. The remodeling simulation can be extended by including more variables and inputs in order to capture complex bone behavior.

## Results

To illustrate the potential of the current mechanobiological remodeling model, some model factors (external load intensity and bone cell rates) were investigated to analyze the impact of these parameters on the remodeling process. The results of these comparative tests were analyzed in terms of the apparent density distribution of the proximal femur.

The remodeling algorithm was implemented in the Abaqus FE code (UMAT routine) to solve the bone remodeling process, incorporating the mechanical and BMU behavior. The iterative process started from constant trabecular and cortical bone densities and ended with variable density at the end of the remodeling process.

**Figure 3:**
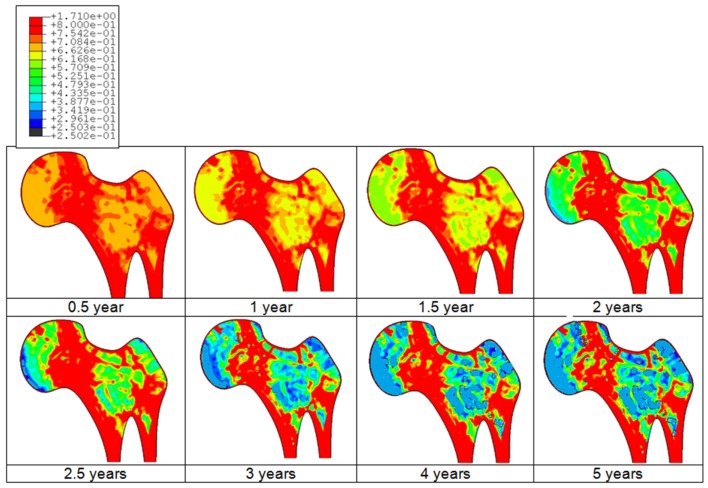
**Predicted bone adaptation sequences in the form of apparent bone density variation in gram per cubic centimeter**.

An example of the bone adaptation sequences of a femur is given in Figure 3. Predicted results indicate that the bone starts the adaptation its density after about 3 months and undergoes continuous adaptation to converge to a steady state after about 4–5 years duration. Indeed, it can be clearly observed that after about 4 years, no additional adaptation can be observed.

### Model validation

Due to lack of specific experimental data related to human femur remodeling process, the complete validation of the current mechanobiological FE model is hard to achieve. Therefore, the obtained results (density distribution) were compared (i) to those obtained by the classic phenomenological remodeling approach of Huiskes (Huiskes et al., 1987; Ruimerman et al., 2005) considered as the gold standard for the simulation of bone remodeling (Cox et al., 2011) and (ii) to experimental histological proximal femur 2D sections from the literature.

**Figure 4:**
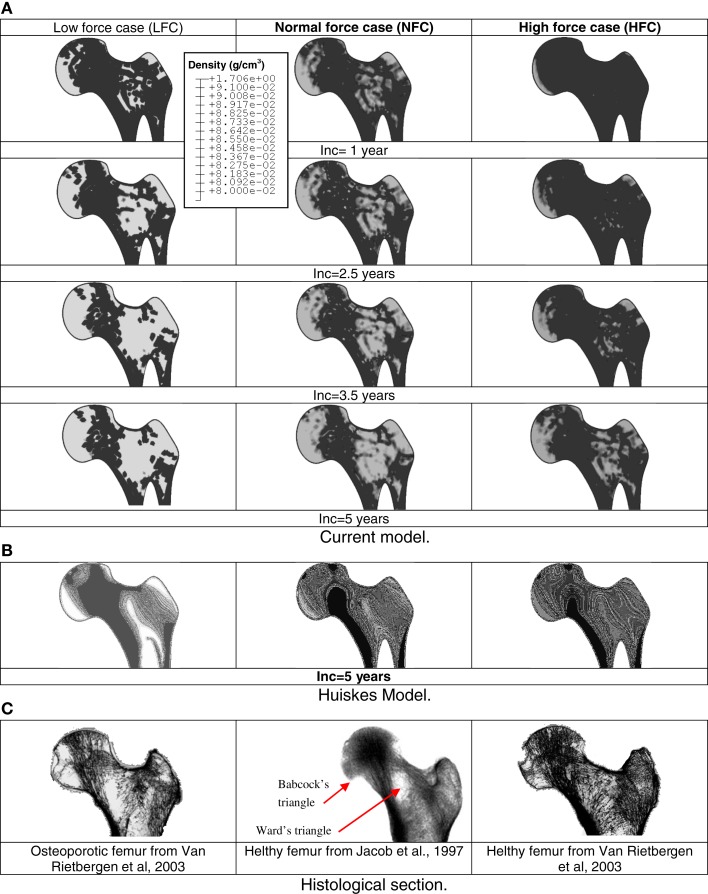
**Sequences of predicted density distributions (gray level) for three different remodeling load levels**. White regions represent low bone density and dark regions represent high bone density. Comparison between current mechanobiological and mechanical models **(A)** prediction based on the current mechanobiological model, **(B)** prediction based on classic phenomenological remodeling approach of Huiskes (Huiskes et al., 1987; Ruimerman et al., 2005). **(C)** Histological proximal femur sections from the literature.

Figure 4 shows the predicted contours of bone density at different sequences for three different remodeling load amplitudes. Bone cell parameters were assumed to be constant (control values). As the focus here is to assess the remodeling of the proximal femur only, the acetabulum was removed (post-processing) from the figures.

Depending on the applied load, in the bone sites where the stimulus was high, the cellular activity generated bone formation by osteoblasts and hence, an increase in density. In the areas where mechanical stimulus was low, the concerned areas were under resorption by osteoclasts or were in a steady state. The model clearly indicated that in the absence of mechanical stimulus, the bone was not completely resorbed and reached a new steady state after about 60% of bone loss.

It can be seen (Figure 4B) that globally; the density distributions predicted by the Huiskes model are similar to those predicted by current model. Nevertheless, the density levels are about 20% lower and the bone sites affected by remodeling predicted by the current model are larger than those calculated by the Huiskes model. These differences can be explained by the facts that: (i) in the early stages of the remodeling process, the current model starts the adaptation process by bone resorption (decrease in density). Therefore, the final results predicted a lower density than that obtained with the Huiskes model, which performs the adaptation process by combining formation and resorption depending on the stimulus level on the bone site. (ii) The model parameters governing the behavior of the osteoblasts and osteoclasts have never been subjected to calibration by experiments.

All main features of femur head density distribution predicted by the current model are more realistic compared to those predicted by Huiskes model. During the bone remodeling process, current model suggests that bone formation and resorption is governed by cells dynamic rather than smoothed adaptive elasticity approaches. Therefore, density heterogeneity and voids (Figure 4A) can be observed to correspond to the experimental profiles (Figure 4C).

### Model parameters sensitivity analysis

**Figure 5:**
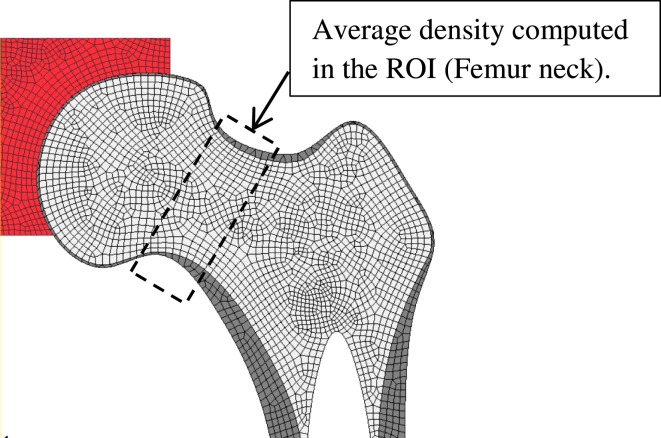
**Region of interest (ROI) where the variation of bone density is computed for the sensitivity analysis**.

The application of the proposed FE remodeling model requires about 30 mechanobiological factors (Table 1), which depend among others on aging, gender, pathologies, drugs intake, etc. Therefore, an SA was performed to investigate the impact of these factors’ sensitivities on the predicted density variation in a selected ROI consisting on the femur neck (Figure 5).

Due to the relative high number of the model 30 factors, a limited preliminary one-factor SA analysis was performed in which only one model parameter value was varied by a given amount while the other parameters were kept at their reference values (Hambli, 2013).

For each parameter change, a remodeling simulation was performed for a duration of 5 years (1825 days) under normal daily loading (see Simulation of Femoral Head Remodeling for details) and the predicted density variation in the femur neck was computed for each simulation. The whole remodeling input parameters listed in Table 1 were each varied by −50 and +50% with respect to their reference values. A single run of the reference parameter values of the model was compared to single runs of the model with each parameter changed individually. SA analysis consisted of 30 total runs with a total computation time about 60 h on a 64 GB computer.

## Discussion

In this study, we have developed a mechanobiological FE model for bone remodeling, which includes a number of relevant mechanical and osteoblast/osteoclast/osteocyte processes and have used this model to address differences in the remodeling behavior of a human proximal femur in disuse or overload and for different bone cell rates. A coupled strain–damage stimulus function was implemented, which controls the level of autocrine and paracrine factors. The cellular behavior is based on Komarova et al.’s dynamic law (2003), which describes the autocrine and paracrine interactions between osteoblasts and osteoclasts and computes cell population dynamics and changes in bone mass at a discrete site of bone remodeling. Therefore, when an external mechanical stress was applied, bone formation and resorption is governed by cells dynamic rather then adaptive elasticity approaches. The remodeling algorithm developed has been applied for different cases (varying remodeling loads and osteoclast/osteoblast growth). The model predicted realistic and plausible results concerning the bone density distribution.

All main features of femur head density distribution are realistic including voids representative of Ward’s and Babcock’s triangles. According to Ward’s classification (Whitehouse and Dyson, 1974), there are different features, which characterize the human proximal femur and among them the so-called Ward’s and Babcock’s triangles. Ward’s triangle represents a central area where trabecular reinforcement is absent or with lower bone mineral densities than other femoral parts in the medulla and in both anterior and posterior walls of the neck. Babcock’s triangle is located between the principal compressive and principal tensile trabecule. In other regions of the head, depending on the remodeling load amplitude, the density decreases gradually from the highest density region to the Ward’s triangle region located close to the femur neutral axis of bending (low strain level). The current mechanobiological algorithm model predicts different bone density distributions depending on the mechanical parameters (applied external loads) and BMU parameters (production and removal rates) in conformity with reported clinical results. Results related to the effects of the BMU rates imply that the remodeling algorithm is sensitive to variation in the production and removal rates of BMUs. It is well-known that the average life span of osteoblasts (≈3 months) exceeds the life span of osteoclasts (≈2 weeks) by a factor close to 6 (Manolagas, 2000). Therefore, the model of BMUs is most sensitive to osteoclast rates, reflecting the fact that osteoclasts are very active in resorbing bone with a lower population number compared to that of osteoblasts. Osteoblasts are much less active and require more time to form bone, as shown by the RS treatment simulation.

The present model incorporates relationships between mechanical stimuli and the osteoclasts’ and osteoblasts’ production of autocrine and paracrine factors through the exponents, *g*12 and *g*21. These relations generated changes in BMU activity, which makes it possible to simulate osteoporosis treatment with hormones, pharmaceutical agents, exercise, and combinations of the three over prolonged periods of time. Such a model would be useful for identifying optimal treatment methodologies as well as changes in bone strength resulting from osteoporosis treatments. Therefore, one of the potential applications of the current model is its ability to investigate the effects of variations in BMU rates variation as a result of a given treatment and dose. By performing iterative simulations on the effects of drug treatments and doses on bone volume, one can predict the optimal treatment strategy to reduce osteoporosis and fracture risk of a specific patient.

### Sensitivity analysis

**Figure 6:**
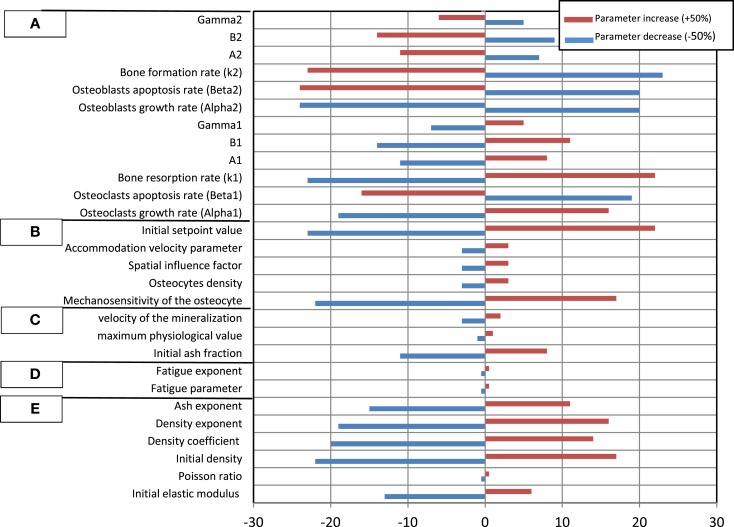
**Relative percentage change of the predicted bone density in the femur neck**. Model parameters were varied between −50 and +50%. **(A)** Osteoblasts and osteoclasts factors, **(B)** transduction factors, **(C)** mineralization factors, **(D)** fatigue damage factors, **(E)** general (mechanical) factors.

The results of the SA are plotted in Figure 6, which shows the impact that a fixed change in each model parameter has on the variation of the bone density in the femur neck.

The results of the SA underscore the influence of each parameter on the resulting bone density. The results indicate that variations of the Young modulus and density growth factors initial density have a significant influence on the bone density variation during the remodeling process. This can be related to the fact that these properties explicitly affect the bone stiffness and hence the bone tissue deformation at a given site. Therefore, the osteocytes detect higher deformation signals, which are transferred ultimately to osteoblasts, which trigger the bone formation process (Adachi et al., 2010; Hambli and Rieger, 2012). Note that the SA analysis showed that the Poisson ratio and the ash exponent parameters have a negligible impact.

Concerning the effect of the fatigue damage process, the SA indicated that for the current simulation boundary conditions (walking cycles during 5 years), the damage factors have a limited impact on the density variation. This can be explained by the relative short duration of the remodeling process (5 years) where the level of the fatigue damage accumulation remains low (critical value where bone damage repair is not reached to trigger osteoclasts to remove the damaged bone).

Among the mineralization process factors, the SA showed that the bone density is sensitive to the initial ash density followed by the velocity of mineralization (Eq. 5). Current model suggests that these factors affect the elastic modulus (Eq. 4), which controls the deformation level of bone.

In the proposed model, the magnitude of the signal received by pre-osteoblasts and pre osteoclasts depends also on the concentration of osteocytes (Eq. 7). This concentration can vary according to the age, sex, type of the bone considered, etc. Present SA results indicated that varying the number of osteocytes with about ±50% generate a slight change on the bone density variation. Nevertheless, the mechanosensitivity factor, the initial set point value, and the accommodation velocity parameter play a significant role on the transduction process and hence, affect significantly the bone remodeling results (Adachi et al., 2010).

Concerning the osteoblasts and osteoclasts activities factors, current SA indicated that the bone density variation is very sensitive to the variation of all osteoblasts and osteoclasts rates. Nevertheless, one can notice that the remodeling process is more sensitive to osteoclast autocrine factors than osteoblast ones. This can be explained by the fact that osteoclasts are very active resorbing cells with a short lifespan, which are recruited and then removed rapidly. In contrast, osteoblasts are much less active with a longer lifespan and a lower concentration changes. Consequently, a greater number of osteoblasts are needed in a single bone remodeling site to counterbalance the resorption of bone by osteoclasts in the same site. The general trend is that (i) an increase in active osteoblast concentration implies an increase in bone formation and therefore prevents bone from resorption and (ii) when varying the cells factors by ±50%, the bone density variation is not symmetric, i.e., the percent of density decrease is higher to density increase.

In summary, the preliminary one-factor SA showed clearly that variation of the remodeling factors, which can be related to aging, gender, pathologies, drugs intake, etc, play a significant role on the remodeling results in terms of bone density. Therefore, the proposed model may be applied to investigate the effects of different diseases and therapies on femur resistance. Therefore, one of the potential applications of the current model is its ability to investigate the effects of variations in BMU autocrine and paracrine rates as a result of a given treatment and dose, calcium–PTH regulation, etc. By performing iterative simulations on the effects of drug treatments and doses on bone volume, one can predict the optimal treatment strategy to reduce osteoporosis and fracture risk of a specific patient.

The current model should be interpreted in accordance with the limiting assumptions contained within the model. The first limitation in the model was that isotropic homogeneous material properties were assigned as an initial condition for the remodeling simulation. A more realistic approach would have been to establish initial conditions based on subject-specific data (DEXA technique, QCT, etc.). In spite of these limitations, as well as the idealized material behavior, the predicted density distribution of the femur still showed many architectural features that are observed clinically. Due to lack of experimental data, the complete validation of the current mechanobiological FE model of bone remodeling is hard to achieve. The second limitation concerns the asynchronous activities of remodeling sites, which will be subjected to expansion in the near future in accordance with additional development and with new experimental results. Also, note that the 2D plane stress assumption is clearly a simplification, since it cannot completely represent a 3D reality. A 2D geometric model does not represent out-of-plane properties. Using 3D FE simulation, Bitsakos et al. (2005) showed that muscle loads can generate significant changes in the periprosthetic response of bone during its remodeling process in the vicinity of external loads. Nevertheless, the hip joint forces used in the current analyses is the greatest load applied on the mediolateral plane of a femur and its magnitude is considerably greater than other loads. Therefore, the 2D femur can be considered as an acceptable representation of 3D remodeling behavior. Third, the present simulations considered the fixed model parameters given in Table 1. These values may be subjected to change due to several factors (disease, age, drugs, gender, bone sites, etc.). Fourth, the present SA considered one-factor analysis was performed in which only one model parameter value was varied by a given amount while the other parameters were kept at their reference values. This simple approach showed in particular that the bone cells rates play significant roles on the bone adaptation process, which may be modulated by specific bone drugs. Nevertheless, for future general SA analysis, it is necessary to consider the full factorial parameters variation simultaneously for different femurs geometries.

## Conflict of Interest Statement

The author declares that the research was conducted in the absence of any commercial or financial relationships that could be construed as a potential conflict of interest.
